# The efficacy of colchicine in preventing atrial fibrillation recurrence and pericarditis post-catheter ablation for atrial fibrillation – A systematic review and meta-analysis of prospective studies

**DOI:** 10.1016/j.ijcha.2024.101466

**Published:** 2024-07-25

**Authors:** Vamsikalyan Borra, Arankesh Mahadevan, Sidhartha Gautam Senapati, Roopeessh Vempati, Vikash Jaiswal, Nithya Borra, Javaria Ahmad, Oscar Rodrigo Zamudio Herrera, Carlos Vergara Sanchez, Tanisha Prasad, Rosy Thachil, Sarju Ganatra, Sourbha Dani

**Affiliations:** a Department of Internal Medicine, The University of Texas Rio Grande Valley, Edinburg, TX, USA; b Department of Internal Medicine, SRM Medical College Hospital and Research Centre, Tamil Nadu, India; c Department of Internal Medicine, Texas Tech University Health Sciences Center, El Paso, TX, USA; d Department of Internal Medicine, Trinity Health Oakland, Pontiac, MI, USA; e Department of Cardiovascular Research, Larkin Community Hospital, South Miami, FL, USA; f Department of Internal Medicine, Sri Venkateswara Medical College, Tirupati, Andhra Pradesh, India; g Divison of Cardiovascular Medicine, Department of Medicine, Lahey Hospital & Medical Center, Burlington, MA, USA; h Department of Cardiology, Marshall University Joan C. Edwards School of Medicine, Huntington, WV, USA; i Department of Cardiology, Mayo Clinic, Jacksonville, FL, USA; j Department of Medicine, Royal College Surgeons, Dublin, Ireland; k Division of Cardiology, Newyork City Health+Hospitals/Elmhurst, Mount Sinai School of Medicine, Queens, NY, USA

**Keywords:** Colchicine, Catheter ablation, Atrial fibrillation, Pericarditis

## Abstract

**Introduction:**

Catheter ablation (CA) initiates a proinflammatory process responsible for atrial fibrillation (AF) recurrence (25–40%) and pericarditis (0.8%). Due to its anti-inflammatory properties, colchicine, a microtubule inhibitor, is explored for the prevention of early AF recurrence and pericarditis after pulmonary vein isolation. We performed a pooled analysis to determine the rates of AF recurrence and pericarditis after CA in patients receiving colchicine.

**Methods:**

A comprehensive literature review was conducted on PubMed and SCOPUS from inception to December 2023 using medical subject headings and keywords, followed by a citation and reference search. We identified prospective studies reporting recurrent AF and pericarditis outcomes after catheter ablation in patients taking colchicine versus placebo. A binary random effects model was used to estimate pooled odds ratios and 95% confidence intervals. Sensitivity analysis was conducted using the leave-one-out method, and heterogeneity was assessed using the *I^2^* statistic.

**Results:**

Of the 958 identified studies, 4 met our inclusion criteria. A total of 1,619 patients were analyzed; 743 received colchicine, and 875 were in the placebo group. Recurrent AF after CA occurred in 192 (29.0 %) of the colchicine group and 318 (39.5 %) of the placebo group. Post-ablation pericarditis occurred in 34 (5.3 %) of the colchicine group and 128 (16.5 %) of the placebo group. Pooled analysis of prospective studies showed that colchicine decreased the odds of recurrent AF [OR: 0.63 (95 % CI: 0.50–0.78), p < 0.01, *I^2^* = 8 %] and post-ablation pericarditis [OR: 0.34 (95 % CI: 0.16–0.75), p < 0.01, *I^2^* = 57 %]. Odds of GI disturbance were increased with colchicine use in our analysis [OR: 2.77 (95 % CI: 1.17–6.56), p = 0.02, *I^2^* = 84 %].

**Conclusion:**

Colchicine use is associated with decreased odds of recurrent AF and pericarditis post-CA from the analysis of prospective studies. These results underscore the potential for colchicine therapy for future exploration with randomized and controlled research with different dosages.

## Highlights

• Recurrent AF after CA occurred in 29.0% of the colchicine group and 39.5% of the placebo group.
• Post-ablation pericarditis occurred in 5.3 % of the colchicine group and 16.5% of the placebo group.
• Pooled analysis of prospective studies showed that colchicine decreased the odds of recurrent AF [OR: 0.63 (95 % CI: 0.50–0.78), p < 0.01, *I^2^* = 8 %] and post-ablation pericarditis [OR: 0.34 (95 % CI: 0.16–0.75), p < 0.01, *I^2^* = 57 %].

## Graphical abstract

**Figure:**
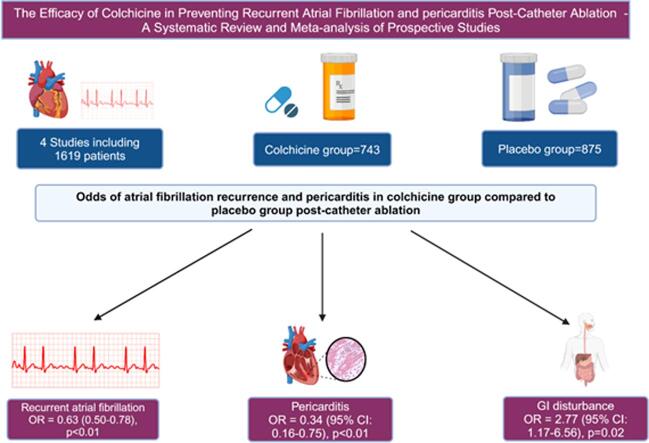

## 1 Introduction

Catheter ablation (CA) for atrial fibrillation (AF) has evolved from a last-resort therapy to a first-line recommendation for improving symptoms and reducing progression to persistent AF [1], [2]. It is also preferred for treating symptomatic patients unable to take anti-arrhythmic therapy and those with heart failure with reduced ejection fraction (HFrEF) [2]. CA, one of the most commonly performed procedures, effectively maintains normal sinus rhythm compared to medical therapy (MT) [1]. Over the past 20 years, it has emerged as a sensible strategy and has significantly advanced with a success rate of 50–80 % [3]. Studies have shown that CA is superior to anti-arrhythmic therapy in reducing AF recurrence and improving Quality of Life (QoL) [4], [5]. Despite effectively treating AF, CA carries few complications [6]. The most common significant complications include vascular access injuries, stroke, cardiac tamponade, and hemothorax [6], whereas minor or less frequent complications include recurrent AF and pericarditis [3].

CA initiates a pro-inflammatory process responsible for AF recurrence (25–40 %) and pericarditis (0.8 %) after ablation [7], [8], [9]. Although not severe, these complications can increase cardiovascular hospitalizations and morbidity. Researchers are exploring colchicine, a microtubule inhibitor, for preventing AF recurrence and pericarditis after CA due to its anti-inflammatory properties [10], [11]. Its reduced cardiovascular side effects make it preferable over corticosteroids or non-steroidal anti-inflammatory drugs [12], [13]. Colchicine is tested in various randomized clinical trials (RCTs) for this purpose, but inconsistent results emerged, likely due to the small sample sizes and colchicine dosage variations [10], [11], [14], [15]. This systematic review and pooled analysis aimed at determining the rates of AF recurrence and pericarditis after CA in patients receiving colchicine compared to those not receiving colchicine based on the data from RCTs and observational studies.

## 2 Methods

**Fig. 1:**
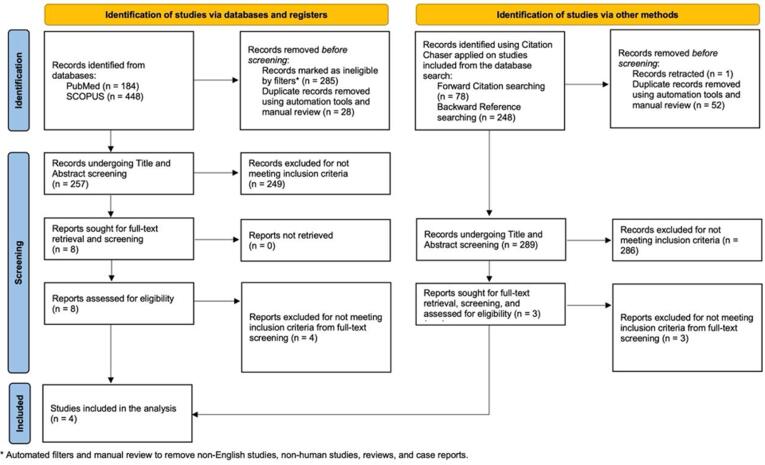
Preferred reporting items for systematic reviews and meta-analyses (PRISMA) chart.

The study's main objective was to explore the effect of colchicine on atrial fibrillation recurrence, post-procedure pericarditis, and gastrointestinal disturbance in the post-cardiac ablation patient cohort. The study was composed according to the Preferred Reporting Items for Systemic Reviews and Meta-Analysis statements (PRISMA) [16] [Fig. 1].

### 2.1 Search strategy and selection criteria

**Fig. 2:**
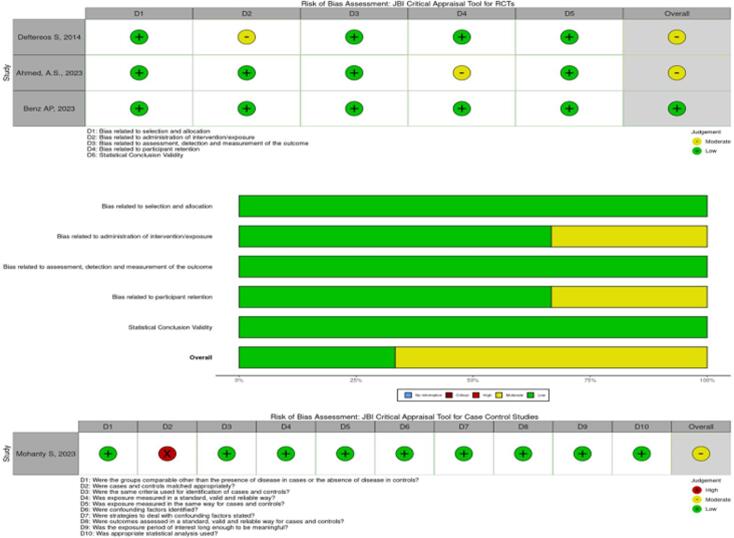
Traffic light plot and summary plot.

A comprehensive literature search was performed by two reviewers (VB and AM) of the PubMed and SCOPUS databases from 2000 through December 2023 using Medical Subject Headings (MeSH) and keywords in the title and body of abstracts. The keywords were combined using appropriate Boolean operators, and the search terms are used (“Atrial Fibrillation”[Mesh] OR “Atrial fibrillation”[all] OR “Supraventricular arrhythmia”[all] OR “AFib”[all] OR “AF”[all]) AND (“Colchicine”[all] OR “Colchicine”[Mesh]). We included studies published in English, prospective studies exploring cohorts with vs. without colchicine use and reporting on outcomes of AF recurrence and post-procedure pericarditis. Articles that were *meta*-analyses, case reports, reviews, animal studies, lacking full-text reports, and not representing the post-cardiac ablation cohort were excluded. Duplicates were manually removed by the reviewer (AM). Two reviewers (VB and AM) independently conducted the title and abstract screening, and consensus resolved disagreement after reviewing the full text of the articles. We have also conducted forward citation and backward reference searches via Citation Chaser for each study included in the final analysis from the database search, screening the results for inclusion in the analysis [17]. We used the JBI critical appraisal tool for cohort studies to assess the quality of eligible studies and illustrated the findings as a composite traffic-light plot [Fig. 2].

### 2.2 Data extraction and analysis

Two reviewers (VR and NB) collected data, verified by reviewer AM, including characteristics of the study population, methodological details, and outcomes. The primary outcomes of interest were (1) the risk of AF recurrence, (2) the risk of post-procedure pericarditis between cohorts with and without colchicine use, and (3) a composite outcome of gastrointestinal adverse events encompassing nausea, vomiting, and diarrhea as reported in the included studies. Our analysis utilized unadjusted odds ratios (OR) with 95 % confidence intervals (CI) to measure the association between colchicine and the odds of outcomes. We assessed publication bias using the Luis Furuya-Kanamori (LFK) index, illustrated in a DOI plot. A binary random effects model estimated the pooled OR for the *meta*-analysis, and the results were presented in forest plots. We used *I^2^* statistics to assess the heterogeneity of effect sizes across different studies. Sensitivity analysis was conducted using the leave-one-out method. A p-value < 0.05 defines statistical significance. We analyzed data using R (version 4.3.1) (The R Project for Statistical Computing, Vienna, Austria). Ethical approval was not required as this study is a *meta*-analysis based on existing research and does not involve primary data collection.

## 3 Results

Our initial search in the PubMed and SCOPUS databases using keywords with Boolean operators revealed 184 and 448 studies, respectively. After removing 285 articles by applying filters and 28 duplicate articles, 257 were eligible for title and abstract screening, out of which we excluded 249 that did not meet inclusion criteria. Subsequently, we assessed the remaining eight articles for eligibility, and after full-text screening, we excluded 4 articles for failing to meet the inclusion criteria. Finally, we included four studies in our analysis [10], [11], [15], [18] (Table 1).

**Table 1:** Overview of included studies.

On the other hand, we also identified 326 articles through forward and backward citation chasing. After removing duplicate and retracted articles, 289 were eligible for screening, but we excluded them because they did not meet the inclusion criteria (Fig. 1).

A total of 1,619 patients were analyzed; 743 received colchicine, and 875 were in the placebo group.

### 3.1 Recurrent atrial fibrillation

**Fig. 3:**
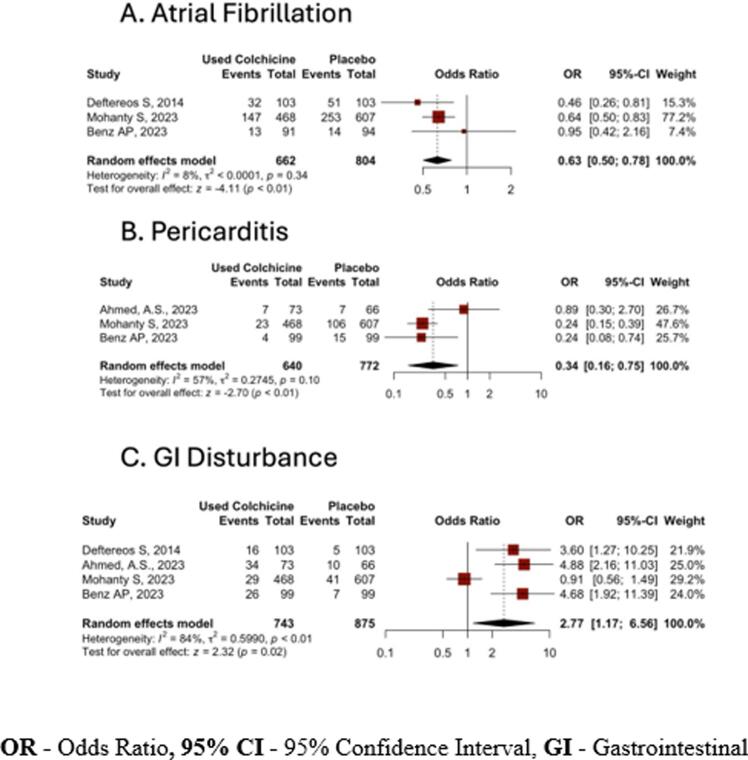
Odds ratio (OR) of outcomes in colchicine compared to placebo group

**Fig. 4:**
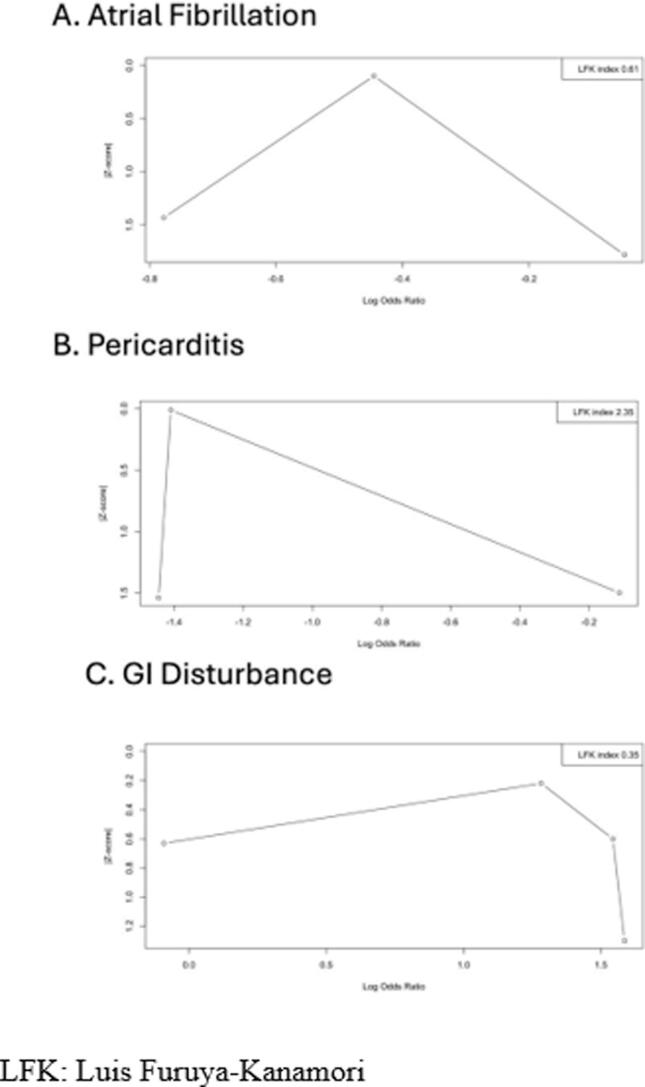
Publication bias.

In our analysis, among the patients treated with colchicine, 192 (29.0 %) developed recurrent atrial fibrillation, while 318 (39.5 %) patients developed recurrent AF among the placebo group. Pooled analysis of prospective studies showed decreased odds of recurrent AF in the colchicine group compared to the placebo group [OR: 0.63 (95 % CI: 0.50–0.78), p < 0.01, *I^2^* = 8 %] with statistical significance (Fig. 3). Publication bias was assessed by the LFK index (0.61), indicating no asymmetry or publication bias, and visualized by DOI plots (Fig. 4).

### 3.2 Post-ablation pericarditis

In our analysis, post-ablation pericarditis occurred in 34 (5.3 %) of those receiving colchicine and 128 (16.5 %) of the placebo group. The pooled analysis of included studies showed decreased odds of post-ablation pericarditis [OR: 0.34 (95 % CI: 0.16–0.75), p < 0.01, *I^2^* = 57 %] with statistical significance (Fig. 3). We assessed publication bias using the LFK index (2.31), indicating mild asymmetry or lesser publication bias, and visualized it with DOI plots (Fig. 4).

### 3.3 Adverse effect: gastrointestinal disturbance

Gastrointestinal (GI) events were reported in 105 (14.1 %) of the colchicine group and 63 (7.2 %) placebo group. Pooled analysis of GI disturbance showed increased odds with colchicine use compared to the placebo group in our analysis [OR: 2.77 (95 % CI: 1.17–6.56), p = 0.02, *I^2^* = 84 %] (Fig. 3). We assessed publication bias using the LFK index (0.35), indicating no asymmetry or publication bias, and visualized it with DOI plots (Fig. 4).

## 4 Discussion

### 4.1 Introduction to colchicine and catheter ablation in AF management

In our analysis of prospective studies, patients receiving colchicine had reduced recurrence of AF and pericarditis and increased GI side effects. CA is a well-established treatment for AF and related symptoms [1]. AF recurrence remains a notable challenge despite the procedure's success, impacting 25–40 % of patients within a year [19]. The pro-inflammatory effects (increase in CRP and IL-6) and oxidative stress (MPO and ROS) intrinsic to ablation have been connected to early AF recurrence and pericarditis [20], [21], [22], [23].

### 4.2 Efficacy of colchicine in reducing AF recurrence and pericarditis

Colchicine, an alkaloid known for its anti-mitotic and anti-inflammatory properties and its efficacy in lowering inflammation, is underscored by studies demonstrating a notable decrease in inflammatory biomarkers (CRP and IL-6) [10], [24]. AF is often preceded by inflammation and structural remodeling of the atria. The inflammatory response can lead to fibrosis and changes in the electrophysiological properties of the atrial tissue, promoting AF [25]. By inhibiting the microtubule polymerization and subsequent inflammatory processes, colchicine can mitigate these effects. This reduction in inflammation and oxidative stress can prevent the structural and electrical remodeling of the atria that predisposes to AF [26].

Furthermore, a few studies have leveraged this anti-inflammatory action to explore colchicine's effectiveness in reducing the likelihood of recurrent AF and pericarditis, with promising results [14], [24]. In our pooled analysis of prospective studies, the colchicine group had a reduced risk of recurrent AF [OR: 0.63 (95 % CI: 0.50–0.78), p < 0.01, *I^2^* = 8 %]. A recent *meta*-analysis, including prospective and retrospective studies, showed no statistical significance for the decrease in atrial fibrillation recurrence and pericarditis post-catheter ablation among those receiving colchicine compared to placebo [27]. In an analysis by Agarwal et al, a decreased odds of recurrent AF (RR 0.76, 95 % CI 0.65 to 0.90, p < 0.01, I2 = 0 %) and no difference in the risk of post-ablation pericarditis (RR 0.51, 95 % CI 0.23 to 1.12, p = 0.10; I2 = 74 %) in patients receiving colchicine as compared with control was observed. Agarwal et al included both observational and RCTs compared to our study, which included only prospective studies [28].

Unlike recent *meta*-analyses and retrospective reviews, our analysis exclusively includes prospective studies. This approach minimizes biases inherent in retrospective data and provides a more robust assessment of colchicine’s efficacy. By concentrating on prospective studies, our review offers a higher level of evidence regarding the impact of colchicine on AF recurrence and post-ablation pericarditis. This approach addresses gaps and limitations in previous *meta*-analyses and retrospective reviews, offering a fresh and robust perspective on colchicine’s clinical utility in AF management. In our analysis, we included only randomized control trials (RCTs) and other prospective studies in addition to including recently published RCT [15], which has not been included in Bulhões et al. The likely hypothesis of our results is that all the prospective studies used a consistent dose of colchicine throughout, contrary to the wide range of doses and the inherent bias associated with retrospective studies. Similar to our results, a *meta*-analysis of RCTs also showed that prophylactic colchicine use reduced the risk of postoperative atrial fibrillation after cardiac surgery [29].

### 4.3 Comparative analysis of colchicine studies

Previously, Deftereos et al. conducted a study evaluating the effects of colchicine (administered at 0.5 mg twice daily for three months) in 161 patients undergoing catheter ablation for atrial fibrillation (AF) [10]. Colchicine initiated on the day of catheter ablation reduced C-reactive protein (CRP) and interleukin-6 levels through day 4, correlating with a significant decrease in early atrial arrhythmia recurrence [10]. Contrarily, Campbell, et al. reported no association between colchicine use and lower AF recurrence or hospitalization rates at 1 year in a matched observational cohort of similar size [30]. Differences in findings compared to the Deftereos et al. study were attributed to variations in patient enrollment and the continued use of antiarrhythmic drugs during the blanking period in the latter study, suggesting a possible lack of efficacy of colchicine in reducing recurrent AF alongside antiarrhythmic drug therapy [30]. Shvartz et al. investigated how effective low-dose, short-term colchicine treatment is for preventing postoperative atrial fibrillation (POAF) in patients undergoing open-heart surgery. The incidence of POAF was 18.6 % in the colchicine group compared to 30.7 % in the placebo group, with decreased odds (OR 0.515; 95 % CI 0.281–0.943; *p* = 0.029) [37]. Since POAF commonly occurs within the first 2–4 days after surgery, starting colchicine before the procedure is essential [31].

### 4.4 Colchicine’s role in pericarditis management

Procedures, such as catheter ablation, trigger an immune response, where neutrophils are considered the primary source of reactive oxygen species (ROS) and myeloperoxidase (MPO) and have been attributed to both atrial fibrillation recurrence and pericarditis due to inflammation [32], [12]. The preferential accumulation of colchicine in neutrophils inhibits directed migration (chemotaxis) and reduces adhesion to inflamed endothelium [10], [11]. The primary treatment for acute pericarditis involves nonsteroidal anti-inflammatory drugs (NSAIDs) and colchicine due to their anti-inflammatory properties [33]. Compared to NSAIDs, colchicine potentially offers enhanced cardiovascular benefits by avoiding involvement in the arachidonic acid pathway, a mechanism utilized by corticosteroids and NSAIDs [11], [13], [12]. In 2015, the European Society of Cardiology (ESC) officially recommended colchicine as a class IA medication for acute and recurrent pericarditis, a growing body of evidence supporting its efficacy and safety in this setting [34]. In our analysis of prospective studies, we found that colchicine was associated with lower odds for post-ablation pericarditis [OR: 0.34 (95 % CI: 0.16–0.75), p < 0.01, *I^2^* = 57 %]. Similar to our analysis, numerous prospective studies and trials have demonstrated that colchicine reduces post-procedure chest pain and shortens hospitalization duration after catheter ablation [9], [35], [36], [37]. This rapid effect aligns with its known anti-inflammatory properties, consistent with its efficacy in preventing and treating pericarditis and post pericardiotomy syndrome following cardiac surgery [37], [38], [39].

### 4.5 Gastrointestinal side effects and limitations of colchicine

Despite being generally safe, its use is constrained by common gastrointestinal side effects, particularly diarrhea [40]. Our analysis underscores the concern for gastrointestinal side effects, revealing significantly higher odds of such disturbances among patients treated with colchicine than those without [OR: 2.77 (95 % CI: 1.17–6.56), p < 0.01, *I^2^* = 84 %]. Colchicine's side effect profile, including diarrhea and nausea [21] and its limited use due to drug interactions [9], raises concerns.

While colchicine’s anti-inflammatory properties hold theoretical promise for AF recurrence and reducing post-ablation pericarditis, they may pose adverse effects. Due to its metabolism and excretion pathways and potential for toxicity, it may be contraindicated in patients with renal or hepatic impairment, gastrointestinal disorders, severe cardiovascular disease, and hematological conditions [41], [42]. Additionally, interactions with CYP3A4 and P-glycoprotein inhibitors can elevate colchicine levels, increasing toxicity risk [42]. Due to the drug's potential adverse effects, patients with neuromuscular disorders, severe infections, or those who are pregnant or lactating require careful consideration and monitoring [43]. Thus, individualized risk assessment is crucial in colchicine therapy.

### 4.6 Study limitations and future directions

This *meta*-analysis has certain limitations, including heterogeneity among the data, a notable scarcity of large-scale randomized controlled trials (RCTs), different dosages and duration of colchicine therapy used in the included studies, and the challenges in the utilization of colchicine due to interactions with a range of medications, particularly antiarrhythmics. The included studies don’t have information about different catheter ablation techniques, type of atrial fibrillation on which AF recurrence can vary, and the complication rates, which can vary based on the ablation technique. More prospective studies are needed, focusing on varying dosages and durations of colchicine to tailor the therapy for patients undergoing CA to prevent recurrent AF and pericarditis.

## 5 Conclusion

Based on our analysis of the prospective studies, colchicine use is associated with a reduced risk of recurrent AF and pericarditis post-CA. The study emphasizes the need for further randomized and controlled research with different dosages and durations of colchicine therapy to tailor the management based on the patient's characteristics.

Ethics approval: Since the data included in this review is available in publicly accessible databases, the IRB review was not mandatory. This review was in accordance with the ethical standards of the institutional and national research committee and with the 1964 Helsinki Declaration and its later amendments or comparable ethical standards.

## CRediT authorship contribution statement

**Vamsikalyan Borra:** Writing – review & editing, Writing – original draft, Data curation. **Arankesh Mahadevan:** Writing – review & editing, Writing – original draft, Software, Conceptualization. **Sidhartha Gautam Senapati:** Writing – review & editing, Writing – original draft. **Roopeessh Vempati:** Writing – review & editing, Writing – original draft. **Vikash Jaiswal:** Writing – review & editing, Writing – original draft. **Nithya Borra:** Writing – review & editing, Writing – original draft, Data curation. **Javaria Ahmad:** Writing – review & editing, Writing – original draft, Data curation. **Oscar Rodrigo Zamudio Herrera:** Writing – review & editing, Writing – original draft, Data curation. **Carlos Vergara Sanchez:** Writing – review & editing, Writing – original draft, Data curation. **Tanisha Prasad:** Writing – review & editing, Writing – original draft. **Rosy Thachil:** Writing – review & editing, Writing – original draft. **Sarju Ganatra:** Writing – review & editing, Writing – original draft, Data curation. **Saurabha S Dani:** Writing – review & editing, Writing – original draft, Data curation, Conceptualization.

## Declaration of competing interest

The authors declare that they have no known competing financial interests or personal relationships that could have appeared to influence the work reported in this paper.
